# The relationship between spexin and liver steatosis in polycystic ovary syndrome: a novel analysis using ultrasound-guided attenuation parameter

**DOI:** 10.1590/1806-9282.20241313

**Published:** 2025-03-31

**Authors:** İzzet Özgürlük, Rasime Pelin Kavak, Berna Turhan, Sümeyya Duran Kaymak, Rabia Şeker, Gülin Feykan Yeğin, İhsaniye Süer Doğan

**Affiliations:** 1 Ministry of Health, Etlik City Hospital, Department of Clinic of Obstetrics and Gynecology – Ankara, Turkey.; 2 Ministry of Health, Etlik City Hospital, Department of Radiology – Ankara, Turkey.; 3 Ministry of Health, Etlik City Hospital, Department of Medical Biochemistry – Ankara, Turkey.

**Keywords:** SPX, Polycystic ovary syndrome, Nonalcoholic fatty liver disease

## Abstract

**OBJECTIVE:**

This study aims to evaluate the relationship between spexin levels and nonalcoholic fatty liver disease in patients with polycystic ovary syndrome.

**METHODS:**

The study included 90 participants, comprising 44 patients diagnosed with polycystic ovary syndrome and 46 age- and body mass index-matched controls. Participants’ spexin, glucose, urea, alanine aminotransferase, aspartate aminotransferase, total cholesterol, low-density lipoprotein, high-density lipoprotein, triglycerides, progesterone, follicle-stimulating hormone, luteinizing hormone, estradiol, total testosterone, free testosterone, and dehydroepiandrosterone sulfate levels were measured. The liver steatosis grading was done using the ultrasound-guided attenuation parameter from General Electric Healthcare. The parameters were evaluated between the groups.

**RESULTS:**

Spexin levels in polycystic ovary syndrome patients were significantly lower compared to the control group (p<0.001). Glucose, triglycerides, luteinizing hormone, total testosterone, free testosterone, and dehydroepiandrosterone sulfate levels were found to be significantly higher in polycystic ovary syndrome patients compared to the control group (p<0.001). High-density lipoprotein levels in polycystic ovary syndrome patients were significantly lower compared to the control group (p<0.001). The prevalence of liver steatosis was notably higher among the polycystic ovary syndrome patients compared to the control group (p<0.001). In patients with polycystic ovary syndrome, spexin levels demonstrated significant negative correlations with body mass index, glucose, triglycerides, luteinizing hormone, total testosterone, free testosterone, and dehydroepiandrosterone sulfate (all p<0.001). A strong negative correlation was observed between spexin levels and liver steatosis grading (p<0.001).

**CONCLUSION:**

Spexin is a coordinator in the metabolic relationship between polycystic ovary syndrome and liver health, suggesting its utility as a biomarker for detecting liver steatosis and related metabolic disturbances in this population.

## INTRODUCTION

Adipose tissue secretes adipokines and growth factors that can modulate the reproductive axis. The regulation of menstruation involves a coordinated interaction between the hypothalamus, anterior pituitary gland, ovaries, and endometrium. During adolescence, menstrual irregularities are common, particularly within the first 2 years post-menarche, due to the immaturity of the hypothalamic–pituitary–ovarian (HPO) axis. This immaturity is characterized by an atypical response of luteinizing hormone (LH) to estradiol. Under normal conditions, the maturation of an ovarian follicle leads to increased estradiol production, which triggers a surge in LH. However, in adolescents, this response may be atypical, with only a weak LH production that fails to generate the peak necessary for ovulation. Furthermore, the insufficient inhibition of follicle-stimulating hormone (FSH) by inhibin can result in an excess of ovarian follicles entering the growth phase, contributing to the underdevelopment of the HPO axis and subsequent menstrual irregularities^1^.

Polycystic ovary syndrome (PCOS) is a prevalent endocrine and metabolic disorder that affects women of reproductive age, characterized by hyperandrogenism, ovulatory dysfunction, and polycystic ovarian morphology. The diagnosis of PCOS is commonly established based on the Rotterdam criteria, which require the presence of at least two of these three features^2^.

The diagnosis of PCOS is primarily clinical, relying on the presentation of characteristic signs and symptoms, particularly menstrual irregularities. The condition encompasses a wide and diverse range of manifestations, including menstrual dysfunction, hyperandrogenism, hirsutism, metabolic disturbances, and infertility. Recent studies have highlighted the role of neuroendocrine mechanisms in the pathogenesis of PCOS, suggesting that increased adipose tissue may impact the HPO axis^1^. This hypothesis has been supported by clinical findings from Tay et al.^3^ The HPO axis is a vital endocrine system that governs female reproductive function. This axis involves a complex interplay between the hypothalamus, pituitary gland, and ovaries, playing a crucial role in regulating the menstrual cycle, ovulation, and overall reproductive health^3^. Disruptions within this system can lead to various reproductive disorders, including PCOS. The hypothalamus produces gonadotropin-releasing hormone (GnRH), which stimulates the pituitary gland to release two key hormones: LH and FSH. In response to GnRH, the anterior pituitary secretes LH and FSH. These hormones are essential for the growth and maturation of ovarian follicles, ovulation, and the production of sex hormones (estrogen and progesterone) by the ovaries. The ovaries, responding to LH and FSH signals, produce estrogen and progesterone. These hormones are critical for regulating the menstrual cycle and maintaining reproductive health. Disruptions in the HPO axis can result in conditions such as PCOS, which is characterized by irregular menstrual cycles, hyperandrogenism, and polycystic ovaries. In PCOS, insulin resistance and hormonal imbalances can interfere with the normal functioning of the HPO axis, contributing to the syndrome's clinical features^1^. The high prevalence of PCOS, combined with its diverse lifelong manifestations, is further complicated by obesity, which exacerbates its clinical symptoms and contributes significantly to the global disease burden. Therefore, early diagnosis of PCOS is crucial for facilitating timely interventions and preventing complications^4^. Long-term weight gain remains a significant health issue for women with PCOS and is a fundamental pathophysiological factor that can worsen the severity of the syndrome.

Beyond its gynecological implications, PCOS is increasingly recognized for its association with various concurrent comorbidities and metabolic disturbances, including insulin resistance, dyslipidemia, and a heightened risk of cardiovascular disease^5^.

Recent research has also highlighted a significant link between PCOS and nonalcoholic fatty liver disease (NAFLD), a condition marked by the accumulation of fat in the liver, as detected by imaging or histological examination, in the absence of other identifiable causes of liver steatosis^6^. NAFLD is considered the most common liver disorder worldwide, and it poses a substantial risk for progressing to more severe liver diseases, such as cirrhosis and hepatocellular carcinoma. Women with PCOS have been found to have a higher predisposition to developing NAFLD compared to their non-PCOS counterparts, likely due to the overlapping metabolic dysfunction characteristic of both conditions^6^.

Spexin (SPX), a novel peptide hormone consisting of 14 amino acids, has recently emerged as a key player in the regulation of metabolic processes. Identified through Markov modeling, SPX is expressed in various tissues, including those of the hypothalamus–pituitary–gonadal axis, suggesting a potential role in both metabolic and reproductive functions^7^.

The physiological significance of SPX in humans, particularly in the context of metabolic diseases and inflammation, has become a focus of growing interest. In particular, the expression of SPX in granulosa cells and its inhibitory effects on steroidogenesis and cell proliferation have been noted, further linking this peptide to the pathophysiology of PCOS^8^.

Given the intertwined metabolic and endocrine abnormalities in PCOS, along with the elevated risk of NAFLD in this population, there is a compelling need to explore the role of SPX in these conditions.

The ultrasound-guided attenuation parameter (UGAP) has recently been introduced as a reliable tool for assessing liver steatosis, offering a noninvasive method to quantify liver fat content and potentially linking it to metabolic markers like SPX^5^.

Understanding the relationship between SPX levels and liver steatosis in PCOS patients could provide novel insights into the mechanisms underlying these coexisting disorders and pave the way for new therapeutic approaches.

Given that SPX has recently been identified as a significant regulator of metabolic and reproductive functions and considering the higher risk of NAFLD among women with PCOS, this study seeks to investigate whether SPX could serve as a potential biomarker for assessing the severity of liver steatosis in this population.

## METHODS

This prospective study was conducted at the Department of Obstetrics and Gynecology between May 1 and July 31, 2024. The approval was granted by the Ministry of Health Etlik City Hospital Clinical Research Ethics Committee (Turkey) (ID number: 2024/368). We obtained written informed consent from all participants.

Ninety subjects were enrolled in this case-control study, comprising 44 patients diagnosed with PCOS and 46 age- and body mass index (BMI)-matched controls with normal menstrual cycles. The diagnosis of PCOS was based on the Rotterdam consensus criteria^2^.

BMI was calculated by dividing weight in kilograms by the square of height in meters (kg/m^2^), and participants were divided into two groups based on their BMI: those with a BMI of less than 25 kg/m^2^ and those with a BMI of 25 kg/m^2^ or higher.

Participants with a history of chronic illnesses, alcohol use, focal liver lesions, and recent hormonal contraception or anti-androgen therapy (within the past 3 months), as well as those who were breastfeeding, pregnant, or on medication for conditions like dyslipidemia, were excluded from the study.

Blood samples were collected from all participants between the 3rd and 5th days of their menstrual cycles and were stored at −80°C until analysis. Tests for glucose, urea, alanine aminotransferase (ALT), aspartate aminotransferase (AST), total cholesterol, low-density lipoprotein (LDL), high-density lipoprotein (HDL), triglycerides, progesterone, LH, estradiol, total testosterone, and dehydroepiandrosterone sulfate (DHEAS) were conducted using Roche Cobas 8000 modular equipment. The assays for glucose, urea, ALT, AST, total cholesterol, LDL, HDL, and triglycerides were performed using spectrophotometry, while LH, DHEAS, progesterone, and total testosterone were measured using electrochemiluminescence immunoassay. Free testosterone levels were determined by radioimmunoassay.

Serum SPX levels were quantified using an enzyme-linked immunosorbent assay (ELISA) kit (Cat. No. E-EL-H5607; Lot: WA15HN2D5311; Elabscience Bionovation Inc.) in accordance with the manufacturer's instructions. The SPX concentrations were determined from the standard curve generated during the assay and were reported in pg/mL. The intra-assay coefficient of variation was calculated to be less than 10%. The functional sensitivity of the assay was 46.88 pg/mL, and the detection range of the kit was 78.13–5,000 pg/mL.

**Figure 1:**
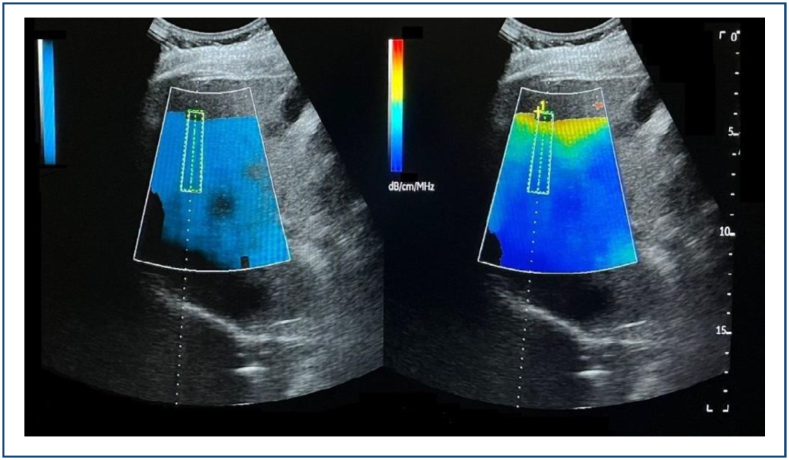
Ultrasound-guided attenuation parameter mode examinations were performed on liver segment V. Regions of interest measuring were placed within the blue color-coded box on the quality map, ensuring the avoidance of bile ducts, vessels, and shading artifacts. The ultrasound system automatically calculates the attenuation coefficient (dB/cm/MHz).

Three experienced radiologists, who were blinded to the clinical status of the participants, conducted the UGAP measurements using a LOGIQ E10 ultrasound machine (General Electric Healthcare) with a convex probe in consensus. UGAP mode examinations were performed on liver segment V, located inferior to the right anterior lobe, while the participant held their breath. Regions of interest measuring were placed within the blue color-coded box on the quality map, ensuring the avoidance of bile ducts, vessels, and shading artifacts. The liver steatosis grading was determined using the General Electric whitepaper guidelines. The ultrasound system automatically calculates the attenuation coefficient (dB/cm/MHz) (Figure 1).

SPX, glucose, urea, ALT, AST, total cholesterol, LDL, HDL, triglycerides, progesterone, FSH, LH, estradiol, total testosterone, free testosterone, DHEAS levels, and liver steatosis grading were evaluated between the groups.

### Statistical method

Descriptive statistics for continuous data were presented as mean±standard deviation, median, and interquartile range (25th–75th percentiles), while categorical data were expressed as frequencies and percentages. The Kolmogorov-Smirnov test was used to assess the normality of data distribution. For comparisons between PCOS patients and the control group, the Student's t-test was applied to normally distributed continuous variables, and the Mann-Whitney U test was used for non-normally distributed variables. For nominal variables, group comparisons were conducted using the chi-square test in contingency tables. Relationships between SPX levels and continuous variables were analyzed using Spearman's correlation coefficient. All statistical analyses were performed using IBM SPSS for Windows, version 20.0 (SPSS Inc., Chicago, IL). p-values less than 0.05 were considered statistically significant.

## RESULTS

The study included a total of 90 participants, comprising 44 patients diagnosed with PCOS and 46 age- and BMI-matched controls.

SPX levels in PCOS patients were significantly lower compared to the control group (p<0.001). Glucose, triglycerides, LH, total testosterone, free testosterone, and DHEAS levels were found to be significantly higher in PCOS patients compared to the control group (p<0.001). HDL levels in PCOS patients were significantly lower compared to the control group (p<0.001). Additionally, the prevalence of liver steatosis was notably higher among the PCOS patients compared to the control group (p<0.001) (Table 1).

**Table 1:** Comparison of parameters and liver steatosis between women with polycystic ovary syndrome patients and control group.

Parameters		PCOS patients (n=44)	Control group (n=46)	p-value
Age (year)		26.59±3.30	25.97±3.61	0.282^a^
Body mass index		24.9 (22.7–27.4)	24.1 (22.0–26.7)	0.187^b^
Body mass index Group c				
	Body mass index<25 kg/m^2^	104 (62.3)	63 (37.7)	0.134^c^
	Body mass index ≥25 kg/m^2^	136 (69.7)	59 (30.3)	
Spexin (pg/mL)		1,323.11 (1,211.23–1,579.0)	3,223.18 (2,568.71–3,777.85)	**<0.001** b
Glucose (mg/dL)		89 (82–105)	80 (77–85)	**<0.001** b
Urea (mg/dL)		28 (24–30)	28 (23–30)	0.761^b^
Alanine aminotransferase (U/L)		28 (26–29)	26.5 (24–29)	0.208^b^
Aspartate aminotransferase (U/L)		28 (26–29)	26.5 (24–29)	0.208^b^
Total cholesterol (mg/dL)		203 (202–204)	203 (203–204)	0.585^b^
Low-density lipoprotein (mg/dL)		132 (131–133)	132 (131–133)	0.673^b^
High-density lipoprotein (mg/dL)		41.5 (40–42)	50 (50–53)	**<0.001** b
Triglycerides (mg/dL)		143 (140–145)	114 (108–120)	**<0.001** b
Progesterone (ng/mL)		1.2 (1.1–1.3)	1.2 (1.1–1.2)	0.990^b^
Follicle-stimulating hormone (mIU/mL)		6.99 (6.89–7.09)	6.99 (6.88–7.10)	0.961^b^
Luteinizing hormone (mIU/mL)		13.70 (13.50–13.97)	8.04 (8.00–8.07)	**<0.001** b
Estradiol (pg/mL)		48 (47–49)	48 (46–51)	0.885^b^
Total Testosterone (ng/dL)		2.86 (2.84–2.88)	1.69 (1.68–1.70)	**<0.001** b
Free Testosterone (pg/mL)		0.83 (0.81–0.83)	0.54 (0.52–0.57)	**<0.001** b
Dehydroepiandrosterone sulfate (μg/dL)		179 (165–183)	153 (152–157)	**<0.001** b
Liver steatosis grading				
	S0	8 (18.2)	34 (73.9)	**<0.001** c
	S1	6 (13.6)	4 (8.7)	
	S2	19 (43.2)	7 (15.2)	
	S3	11 (25.0)	1 (2.2)	

PCOS: polycystic ovary syndrome.

a Data are presented as mean±standard deviation and Student's t-test.

b Data are presented as median (IQR) (25–75%), and the Mann-Whitney U test.

c Chi-square test. The bold values in the table are blood values.

In patients with PCOS, SPX levels demonstrated significant negative correlations with BMI, glucose, triglycerides, LH, total testosterone, free testosterone, and DHEAS (all p<0.001). Additionally, a strong negative correlation was observed between SPX levels and liver steatosis grading (p<0.001) (Table 2).

**Table 2:** Correlations between the spexin levels, parameters, and liver steatosis grading in polycystic ovary syndrome patients and the control group.

	PCOS patients (n=44)	
	Spexin (pg/mL)	
	r*	p-value
Age	0.293	0.054
Body mass index	**-0.691**	**<0.001**
Glucose (mg/dL)	**-0.719**	**<0.001**
Urea (mg/dL)	**-0.380**	0.011
Alanine aminotransferase (U/L)	0.151	0.326
Aspartate aminotransferase (U/L)	0.151	0.326
Total Cholesterol (mg/dL)	0.117	0.451
Low-density lipoprotein (mg/dL)	-0.017	0.915
High-density lipoprotein (mg/dL)	0.077	0.620
Triglycerides (mg/dL)	**-0.735**	**<0.001**
Progesterone (ng/mL)	0.062	0.690
FSH (mIU/mL)	-0.130	0.399
LH (mIU/mL)	**-0.680**	**<0.001**
Estradiol (pg/mL)	0.125	0.418
Total Testosterone (ng/dL)	**-0.904**	**<0.001**
Free Testosterone (pg/mL)	**-0.742**	**<0.001**
Dehydroepiandrosterone sulfate (μg/dL)	**-0.513**	**<0.001**
Liver steatosis grading	**-0.924**	**<0.001**

PCOS: polycystic ovary syndrome.

* Spearman's correlation coefficient. The bold values in the table are blood values.

## DISCUSSION

The findings of this study highlight a novel association between SPX levels and liver steatosis in PCOS patients, suggesting that SPX may play a role in metabolic regulation. However, the complex pathophysiology of PCOS extends beyond this single biomarker, involving hormonal imbalances, metabolic dysfunction, and inflammatory processes that affect various tissues, including the endometrium.

Recent studies have underscored the influence of metabolic and hormonal factors on endometrial quality and the effectiveness of hormonal treatments. Baracat et al.^9^ reported that despite progesterone treatment, histomorphometric abnormalities in the endometrium persisted in PCOS patients, and these abnormalities were associated with elevated androgen and insulin levels. This suggests that standard hormonal treatments, such as progesterone therapy, may be less effective in PCOS patients with underlying metabolic disturbances, pointing to the need for integrated treatment approaches that address both metabolic and hormonal pathways. For example, combining insulin-sensitizing agents with hormonal therapies might improve endometrial health and fertility outcomes in these patients.

Additionally, research by Giordano et al.^10^ demonstrated that metabolic and hormonal variables, including hyperinsulinemia and hyperandrogenemia, influenced the expression of immune protein biomarkers in the endometrium of women with PCOS. These findings suggest a complex interplay between metabolic dysfunction and immune regulation, which may further exacerbate endometrial abnormalities and contribute to poor reproductive outcomes. Clinically, this highlights the importance of early intervention to manage insulin resistance and hyperandrogenism to prevent the development of chronic endometrial alterations and improve reproductive health in PCOS patients.

Given the multifactorial nature of PCOS, future therapeutic strategies should consider not only the correction of hormonal imbalances but also the management of metabolic and inflammatory pathways. Addressing these interconnected factors could improve clinical outcomes, including enhanced endometrial receptivity, reduced risks of metabolic complications, and improved fertility outcomes. The results of this study add to the growing body of research on PCOS by suggesting that novel biomarkers like SPX might be pivotal in understanding and managing the metabolic aspects of this syndrome. SPX could potentially serve as a biomarker for identifying patients at higher risk of metabolic complications, allowing for more tailored and comprehensive management strategies.

This study is the first to demonstrate a negative correlation between SPX levels and liver steatosis, as measured by UGAP, in patients with PCOS. These findings suggest that lower serum SPX levels with PCOS patients are associated with greater liver steatosis, indicating that SPX may have a critical role in modulating metabolic processes related to lipid storage and distribution. While the precise mechanisms through which SPX influences liver steatosis remain unclear, several potential pathways are hypothesized.

SPX is believed to be integral to lipid metabolism. One of the key contributors to liver steatosis is the enhanced uptake of long-chain fatty acids by hepatocytes. In vitro studies have shown that SPX reduces the uptake of these fatty acids by adipocytes^11^. Moreover, in animal models of diet-induced obesity, treatment with synthetic SPX has consistently led to significant weight loss, potentially due to its inhibitory effects on fatty acid uptake in hepatocytes. In vivo studies have demonstrated that a four-week course of SPX treatment can decrease liver lipid content by 60%^12^.

SPX may also interact with other key metabolic hormones, including androgens and insulin. Research by Kim et al.^13^ has shown that women with PCOS exhibit a higher prevalence of ultrasound-diagnosed NAFLD compared to non-PCOS counterparts (5.5 vs. 2.8%), with hyperandrogenemia playing a significant role in this association. Similarly, Jones et al.^14^ reported that women with PCOS and hyperandrogenism had significantly greater intra-hepatic fat content than normo-androgenic PCOS women and healthy controls, even when matched for age, BMI, and waist circumference (mean hepatic fat content: 12.9 vs. 0.6 vs. 1.9%, respectively).

In PCOS, elevated androgen levels can contribute to increased lipid accumulation and metabolic disturbances. Gu et al.^15^ observed lower circulating SPX levels in adults with type 2 diabetes mellitus, with significant negative correlations between circulating SPX and blood glucose levels. Insulin resistance, a common feature of PCOS, can stimulate increased ovarian androgen production, leading to hyperandrogenemia. This hormonal imbalance exacerbates liver fat deposition and further aggravates insulin resistance, creating a vicious cycle that worsens both PCOS and NAFLD^16^.

SPX has also been implicated in the regulation of insulin sensitivity. Insulin resistance, frequently observed in PCOS, can enhance lipogenesis and reduce fatty acid oxidation in the liver. Low levels of SPX may aggravate insulin resistance, thereby promoting liver fat accumulation.

Furthermore, low-grade chronic inflammation is a recognized component of PCOS pathogenesis. This inflammatory state is thought to play a role in the development of insulin resistance, which is a common feature in many women with PCOS, further complicating their metabolic health^4^. In addition, there is emerging evidence suggesting that SPX may possess anti-inflammatory properties^7^. In a mouse model of type 2 diabetes, SPX treatment has been shown to reduce inflammation by decreasing tumor necrosis factor-alpha and interleukin-6 levels in both serum and liver tissues^17^. Reduced levels of SPX could lead to increased inflammation and oxidative stress, which in turn may promote liver fat accumulation. Moreover, inflammatory cytokines can induce insulin resistance, further contributing to the progression of NAFLD in patients with PCOS.

### Limitations

This study has several limitations. The relatively small sample size and single-center design may limit the generalizability of the findings. Additionally, liver biopsies, the gold standard for NAFLD diagnosis, were not performed. While SPX levels were the focus, other relevant biomarkers such as insulin and inflammatory markers were not assessed, which could have provided a more comprehensive metabolic profile.

## CONCLUSION

To the best of our knowledge, this study is the first to report a negative correlation between serum SPX levels and liver steatosis in PCOS patients, assessed using UGAP. These findings highlight the potential role of SPX as a coordinator in the metabolic relationship between PCOS and liver health, suggesting its utility as a biomarker for detecting liver steatosis and related metabolic disturbances in this population.

## INFORMED CONSENT

We obtained written informed consent from all participants.

## ETHICAL APPROVAL

This study was performed in line with the principles of the Declaration of Helsinki. The approval was granted by the Ministry of Health Etlik City Hospital Clinical Research Ethics Committee (Turkey) (ID number: 2024/368).
